# Benchmarking public large language model responses to patient-facing inflammatory bowel disease questions: informational quality, transparency proxies, and readability

**DOI:** 10.3389/fpubh.2026.1810358

**Published:** 2026-04-10

**Authors:** Xiaoyue Wang, Chengguang Yin, Haiyang He, Jinjiao Guo, Xueting Fu, Feihu Bai

**Affiliations:** 1 Department of Gastroenterology, The Second Affiliated Hospital of Hainan Medical University, Haikou, China; 2 Department of Cardiology, The Second Affiliated Hospital of Hainan Medical University, Haikou, China

**Keywords:** benchmarking, generative artificial intelligence, health literacy, inflammatory bowel disease, large language models, patient-facing, readability, transparency

## Abstract

**Background:**

Patient-facing large language model (LLM) outputs for inflammatory bowel disease (IBD) must be decision-relevant, readable, and verifiable.

**Methods:**

In a cross-sectional benchmark using a guideline-derived question set, five publicly available LLMs provided answers to 20 single-intent patient IBD questions, mapped to prespecified decision-critical domains across the care pathway (100 model–question responses). Queries were conducted from January 17–24, 2026, *via* official web interfaces under default settings (privacy mode; new chat per prompt). Two blinded raters evaluated informational quality and completeness (using DISCERN, EQIP, and the Global Quality Scale), transparency proxies (based on JAMA benchmark criteria), and readability through the Automated Readability Index, Flesch Reading Ease, Gunning Fog Index, Flesch–Kincaid Grade Level, Coleman–Liau Index, and SMOG. Overall differences were assessed using within-question paired Friedman tests with Holm adjustment, and effect size was quantified with Kendall's W.

**Results:**

Interrater agreement was high [DISCERN ICC(A,1) = 0.842; EQIP ICC(A,1) = 0.760; GQS weighted κ = 0.812; JAMA weighted κ = 0.936]. Median DISCERN scores ranged from 43.5 to 57.5, and EQIP scores ranged from 67.5 to 77.5, while transparency remained limited (JAMA median 0–1/4). Readability consistently failed to meet patient targets, with grade-level indices exceeding sixth grade and Flesch Reading Ease medians ranging from 15 to 36 (compared to a target of ≥80 for “easy” readability). All 10 outcomes varied significantly across models (Holm-adjusted *P* < 0.001; *W* = 0.238–0.702).

**Conclusion:**

Under default settings, publicly available LLMs exhibit variable informational quality for IBD but consistently poor transparency and readability. Patient-facing deployment should mandate provenance, currency, and disclosure fields, as well as outputs targeted to appropriate grade levels.

## Introduction

Patients with inflammatory bowel disease (IBD) routinely face decisions under uncertainty—such as determining whether symptoms require urgent evaluation, interpreting monitoring tests, deciding when corticosteroids are appropriate, and assessing the benefits and risks of long-term immunosuppressive therapies (1, 2). In this context, many patients seek timely, conversational health information outside of clinical encounters through online resources, including generative artificial intelligence (AI) tools (3). However, accessibility does not equate to quality: to facilitate safe decision-making, patient-facing content must be accurate, sufficiently complete, readable, and accompanied by verifiability cues that allow users to assess credibility and currency (4).

Large language models (LLMs) have the capability to generate fluent, structured responses to medical questions, prompting interest in their potential as scalable adjuncts to patient education (5). Nevertheless, published evaluations have shown considerable variability in performance and persistent safety-related limitations (6). In cross-sectional testing, LLM answers to physician questions demonstrated only moderate accuracy and reliability, with error rates increasing for more complex questions (7). In settings involving patient queries, chatbot responses may be perceived as more empathetic and of higher quality than physician replies, yet perceived quality does not guarantee clinical correctness or completeness for decision-making (8). More broadly, both narrative and systematic reviews emphasize that outputs are influenced by training data, prompting context, and model updates. Moreover, performance on benchmark tasks may not translate into reliable patient education in real-world use (9, 10).

A central concern for patient-facing deployment is transparency (11). High-quality patient information should communicate sources, conflicts of interest, currency, and pathways to authoritative follow-up. In the absence of these elements, users have limited means to verify claims or contextualize uncertainty. As a result, even minor factual errors or omissions can be difficult to detect, correct, or appropriately weigh, raising the risk of misguided self-management. LLMs often provide minimal provenance cues and may generate plausible but unverifiable statements without citations or time stamps (12). Evidence also indicates that citation behaviors can be unreliable: LLMs may fabricate references during literature-style queries, and comparative studies have documented non-negligible hallucination rates and reference inaccuracies relevant to evidence-based health communication (13, 14). Acknowledging the heterogeneity in chatbot evaluations and the need for methodological rigor, recent reporting guidance (the chatbot assessment reporting tool [CHART]) stresses the importance of standardized disclosure of model configuration, prompting, outcome definitions, and reproducible testing as prerequisites for interpretable health-advice studies (15, 16).

These challenges may be particularly significant in the context of IBD. The disease course is chronic and heterogeneous, with management increasingly involving a broad range of biologic and small-molecule therapies, along with risk-mitigation strategies (e.g., infection screening, vaccination) and well-defined thresholds for urgent evaluation. Additionally, conventional online IBD patient education resources often exceed recommended readability targets, which limits accessibility and may exacerbate disparities in comprehension and self-management (17). Early studies on IBD-focused LLMs indicate their potential utility but also reveal several limitations, including variable completeness, inconsistent alignment with evidence-based guidelines, and insufficient attention to verifiability and readability as co-primary objectives (18–20). Overall, the field lacks sufficiently auditable, evidence-anchored, and between-model comparisons that quantify patient-facing informational quality alongside transparency proxies and readability at the response level.

To address this gap, this study conducted a cross-sectional evaluation of responses generated by five publicly available LLMs to a standardized, guideline-derived set of 20 single-intent, patient-facing IBD questions covering key decision points across the care pathway. Specifically, this study assessed the quality and completeness of the responses using established patient-information instruments (DISCERN and EQIP) and global quality ratings (GQS), evaluated transparency signals against the JAMA benchmark criteria, and quantified readability using six standard formulas. Our hypotheses were as follows: (1) overall performance would differ significantly across models, (2) transparency proxies would remain consistently weak, and (3) readability would often exceed recommended patient-facing targets—potentially reflecting a trade-off between informational depth and accessibility.

## Methods

### Study design and overview

This cross-sectional evaluation compared the quality, transparency, and readability of LLM responses to a standardized, guideline-derived set of patient-facing IBD questions. The unit of analysis was the model–question response. Five LLMs were queried with 20 questions, resulting in 100 responses for evaluation (Table 1).

**Table 1:** Guideline-derived patient-facing question set for IBD.

Question no.	Guideline-derived patient-facing question
1	What is the difference between ulcerative colitis and Crohn's disease?
2	Which tests are used to diagnose IBD?
3	How can I tell if my IBD is active or in remission?
4	How is IBD monitored over time?
5	Which symptoms mean I should seek urgent or emergency care in IBD?
6	What treatments are used for a severe ulcerative colitis flare that requires hospitalization?
7	When is surgery recommended for Crohn's disease?
8	What is the usual first-line treatment for mild-to-moderate ulcerative colitis?
9	What is the usual first-line treatment for mild-to-moderate Crohn's disease?
10	When are corticosteroids used in IBD?
11	Why are corticosteroids not meant for long-term use in IBD?
12	When should biologics or small-molecule drugs be started in IBD?
13	What are the options if a biologic stops working in IBD?
14	What infection screening is needed before starting immunosuppressive treatment in IBD?
15	Which vaccines are recommended for people with IBD on immunosuppression?
16	What should I do if I develop fever while on immunosuppressants in IBD?
17	How often do I need colonoscopy to screen for colorectal cancer in colonic IBD?
18	Can IBD medicines be continued during pregnancy and breastfeeding?
19	Which nutrition or vitamin/mineral deficiencies should be checked in IBD?
20	How is iron-deficiency anemia treated in IBD?

The 20 questions are single-intent, patient-facing items covering the IBD care pathway. These questions were derived from PubMed-indexed clinical practice guidelines and consensus statements, with the corresponding guideline mapping and anchor PMIDs provided in Supplementary Table S1.

IBD, inflammatory bowel disease; UC, ulcerative colitis; ASUC, acute severe ulcerative colitis.

### Guideline corpus (PubMed-indexed only) and a priori directions

To ensure an auditable and clinically verifiable question source, the evidence base was limited to PubMed-indexed guidelines and consensus statements (21–35). Both Chinese and international guideline anchors, the nine prespecified patient decision-critical directions, and the complete question-to-guideline module mapping are outlined in Supplementary Methods and Supplementary Table S1. The final 20-item question set is shown in Table 1. Questions were drafted in English to maximize cross-model comparability and minimize translation-induced artifacts in readability metrics. Language generalizability is discussed in the Discussion section.

### Patient question set development and mapping

Two investigators independently reviewed the recommendation statements, algorithms, and summary tables from the guideline corpus, extracting candidate decision nodes within each prespecified direction. Candidate topics were then converted into single-intent, patient-focused English questions (one concept per item, minimal jargon, no multipart questions), with duplicates or near-duplicates removed. The final guideline-derived question set consisted of 20 single-intent, patient-facing IBD questions covering the care pathway (Table 1). Each question was mapped *a priori* to at least one Chinese guideline module and one international guideline module, where applicable; mapping was documented in Supplementary Table S1, and disagreements were resolved by consensus.

### Large language models and query procedure

Five publicly available LLMs were evaluated, with model versions and their respective public release dates as follows: ChatGPT-5.2-Thinking (OpenAI; released December 11, 2025), DeepSeek-V3.2 (DeepSeek; released December 1, 2025), Gemini 3 Pro (Google; released November 18, 2025), Grok 4.1 Thinking (xAI; released November 17, 2025), and Qwen3-Max (Alibaba; released September 24, 2025).

All queries were conducted between January 17, 2026, and January 24, 2026, in Haikou, China, using Google Chrome and the official web interfaces under default settings (no fine-tuning, no APIs, and no external tools beyond standard product behavior). For each model–question pair, we retained the first complete response returned by the default web interface and did not regenerate outputs to sample within-model stochastic variability. Sessions were conducted in browser privacy mode (incognito/private browsing) while logged in. No APIs or external integrations were used, and all models were queried under default web-interface settings. Personalization settings were not modified intentionally. Although accounts were logged in, each prompt was submitted in a fresh chat, and browser privacy mode was employed to prevent any carryover between sessions. User memory or history features were not enabled or configured, aiming to minimize conversational carryover rather than claiming complete elimination of platform-level personalization.

All questions were entered verbatim (without rephrasing, additional context, or system prompts) to simulate novice user interactions. To further reduce carryover and personalization, each prompt was submitted in a new interaction context (one new chat per question), and a new session was initiated for each model. Browser cache was cleared after each model session. All queries were performed on the same device using Google Chrome.

Refusals and technical failures were handled using an *a priori* defined rule set. A refusal was defined as an output that explicitly declined to answer due to safety/policy constraints or otherwise provided no substantive content addressing the prompt. Any refusal/safety-policy response was retained as returned and was not regenerated. Refusals were assigned the minimum possible value for each instrument to reflect zero informational content (DISCERN total = 16/80; EQIP = 0/100; GQS = 1/5; JAMA = 0/4). For transient technical errors that produced a blank output or system error (i.e., no answer content), the same prompt was resubmitted until a non-blank response was returned; resubmission was not performed on the basis of response quality.

### Outcome measures

Reliability Assessment: Two board-certified gastroenterologists with over 10 years of clinical experience (blinded to model sources) independently evaluated all responses using four established tools. In the case of disagreement, a third gastroenterologist with equivalent qualifications served as the final adjudicator (Table 2 footnotes). The four reliability instruments used were the DISCERN instrument, Ensuring Quality Information for Patients (EQIP), the Global Quality Scale (GQS), and the Journal of the American Medical Association (JAMA) benchmark criteria.

**Table 2:** Interrater agreement for primary outcomes (*N* = 100 model–question units).

Outcome (scale)	Agreement statistic	Value
DISCERN total (16–80)	ICC(A,1), 2-way random, absolute agreement	0.842
EQIP total (%Yes; 0–100)	ICC(A,1), 2-way random, absolute agreement	0.760
GQS (1–5)	Weighted Cohen's κ (quadratic)	0.812
JAMA total (0–4)	Weighted Cohen's κ (quadratic)	0.936

Interrater agreement was assessed across 100 model–question units (5 models × 20 questions). For continuous total scores (DISCERN total and EQIP total), ICC(A,1) (two-way random-effects, absolute agreement) was used. For ordinal ratings (GQS and JAMA total scores), weighted Cohen's κ (quadratic weights) was applied. All ratings were conducted independently, and agreement statistics were calculated prior to resolving any discrepancies.

Blinding and Data Handling: All model outputs were exported immediately after generation and compiled into a standardized, anonymized rating workbook. Model names, branding elements, and interface metadata were removed; each response was assigned a random alphanumeric ID, and the response order was randomized within each question block. Raters evaluated responses offline and were only given access to the question prompt and the corresponding response text.

Rater Training and Calibration: Prior to formal scoring, the two raters completed a structured calibration session led by the senior investigator to harmonize their interpretation of the DISCERN, EQIP, GQS, and JAMA items. EQIP calibration included prespecified item-level decision rules tailored to short-form LLM responses (Supplementary Table S2) to standardize how context-dependent items (e.g., action guidance, currency, and text-only format aids) were scored. This session included (1) a review of instrument definitions and scoring anchors from the original publications; (2) joint discussions of common edge cases (e.g., generic safety disclaimers, implicit vs. explicit sourcing, and partially answered prompts); and (3) a pilot scoring exercise on a small set of non-study responses to align scoring thresholds and documentation practices. The calibration procedure and prespecified decision rules were consistently applied throughout the study.

DISCERN: DISCERN was used to assess the quality of information regarding “treatment choices” in patient education materials.36 It consists of 16 items divided into three sections: reliability of information (items 1–8), quality of information on treatment choices (items 9–15), and an overall rating (item 16). Each item is scored from 1 to 5, with a total score range of 16–80 (36). Higher scores indicate better overall quality. For this study, the following score ranges were defined: 16–26 = “very poor,” 27–38 = “poor,” 39–50 = “fair,” 51–62 = “good,” and 63–80 = “excellent.” Because the prompt set covered decision-critical questions across the IBD care pathway (diagnosis, monitoring, urgent triage, prevention/health maintenance, and treatment), we prespecified that DISCERN Section 2 (items 9–15) would be operationalized as the quality of information on management choices relevant to the prompt, rather than being restricted to pharmacologic or procedural therapies. For non-treatment prompts, “choices” included (as applicable) diagnostic test options, monitoring approaches, red-flag thresholds and care-seeking pathways, preventive interventions (e.g., vaccination or infection screening prior to immunosuppression), and escalation/next-step options. No DISCERN items were treated as “not applicable”; if an item was not addressed in a response, raters assigned a score of 1 (lowest anchor). Item-level definitions and scoring decision rules are provided in Supplementary Table S3 (37).

EQIP: EQIP (ensuring quality information for patients) was selected because it evaluates cross-cutting quality attributes of patient-facing health information (e.g., attribution/provenance, evidentiary framing, clarity, actionability, balance, and presentation), and is therefore applicable to our full set of 20 single-intent IBD questions spanning the care pathway (diagnosis, monitoring, red flags/triage, treatment, prevention, and decision support). EQIP was applied to every model–question response (*N* = 100). All 20 EQIP items were retained and scored as binary Yes (1)/No (0); no items were omitted. To preserve comparability across responses and models, we did not use a “Not applicable” category (i.e., the denominator remained 20 for all responses). Items were scored Yes only when explicitly satisfied in the response; otherwise, they were scored No. Item-level operational definitions and prespecified decision rules for adapting EQIP to short-form chatbot outputs (including context-dependent items such as action guidance, currency, and text-only “visual aids” such as tables/checklists) are provided in Supplementary Table S2. EQIP totals were calculated as (%Yes × 100) = (sum of Yes / 20) × 100 (38). Higher scores indicate better material quality (39).

GQS: The Global Quality Scale (GQS) is a single-item, 5-point Likert scale used to rate the overall quality of patient-facing health information. To improve interpretability and reproducibility, we prespecified anchor-point definitions for each category and applied them uniformly across all responses (Supplementary Table S4). Scores were assigned as follows: 1 = Very poor: Inaccurate, misleading, not helpful; 2 = Poor: Limited accuracy, important gaps; 3 = Fair: Mixed quality; some useful info; 4 = Good: Mostly accurate, useful and organized; 5 = Excellent: High-quality, comprehensive, helpful to patients. These anchors were used during rater calibration and throughout scoring. GQS calibration included review of prespecified anchor definitions and exemplar responses (Supplementary Table S4) to standardize score assignment across raters.

JAMA Benchmark Criteria: JAMA criteria focus on the standardization and transparency of online health information, including four items: authorship, attribution of sources and references, disclosure of conflicts of interest (or sponsorship/advertising), and currency. Each item is scored “Yes/No” (1/0), for a total score of 0–4. Because JAMA was originally developed for static websites, we applied it here as a transparency proxy to quantify whether default, one-shot LLM response text spontaneously provides verifiability cues. To ensure cross-platform comparability, scoring was restricted to the response text only; interface-level disclosures outside the response were not captured and are discussed as a limitation. Higher scores indicate better performance in information transparency and standardization (40).

Readability Assessment: Responses were analyzed using six standard readability formulas, which were calculated *via* an online tool (readabilityformulas.com):

Automated Readability Index (ARI) (41):


$$\begin{array}{l} 4.71\left(\frac{\text{characters}}{\text{words}}\right)+\;0.5\;\left(\frac{\text{words}}{\text{sentences}}\right)\;-\;21.43 \end{array}$$

Flesch Reading Ease Score (FRES) (42):


$$\begin{array}{l} 206.835-\;1.015\left(\frac{\text{words}}{\text{sentences}}\right)\;-\;84.6\left(\frac{\text{syllables}}{\text{words}}\right) \end{array}$$

Gunning Fog Index (GFI) (42):


$$\begin{array}{l} 0.4\left[\left(\frac{\text{words}}{\text{sentences}}\right)\;+\;100\left(\frac{\text{complexwords}}{\text{words}}\right)\right] \end{array}$$

Flesch-Kincaid Grade Level (FKGL) (42):


$$\begin{array}{l} 0.39\left(\frac{\text{words}}{\text{sentences}}\right)\;+\;11.8\left(\frac{\text{syllables}}{\text{words}}\right)\;-\;15.59 \end{array}$$

Coleman-Liau Index (CL) (42):


$$\begin{array}{l} 5.89\left(\frac{\text{characters}}{\text{words}}\right)\;-\;0.3\left(\frac{\text{sentences}}{\text{words}}\right)\;-\;15.8 \end{array}$$

Simple Measure of Gobbledygook (SMOG) (42):


$$\begin{array}{l} 1.430\times \sqrt{\text{polysyllables}\times \frac{30}{\text{sentences}}}\;+\;3.1291 \end{array}$$

The readability scores were compared to the sixth-grade readability level recommended by the American Medical Association and the National Institutes of Health (43). The acceptable readability level in the FRES formula was ≥80.0, while for the other five formulas, it was <6.

### Statistical analysis and visualization

All statistical analyses were conducted using IBM SPSS Statistics 29.0 and R (version 4.3.2). Descriptive statistics are reported as mean ± SD and median [Q1, Q3] per model (*n* = 20 question-level responses per model). Overall between-model differences were assessed using the Friedman test (within-question paired; *k* = five models), with Kendall's W used as an effect size. Holm adjustment was applied across the 10 outcomes. For pairwise comparisons, Wilcoxon signed-rank tests (two-sided; zero differences excluded) were performed, with Holm adjustment applied within each outcome across the 10 pairwise model comparisons. Effect size r, the rank-biserial correlation computed from signed-rank sums (positive indicates the first-listed model scores higher), was reported. All tests were two-sided, with statistical significance set at *P* < 0.05 after multiplicity adjustments.

For min–max normalized heatmap, metric-specific min–max normalization (0–1) was applied, and readability indices where lower values indicate easier readability (ARI, GFI, FKGL, CL, and SMOG) were direction-aligned before normalization so that higher values indicated easier readability. FRES was retained in its original direction (higher = easier readability).

For composite trade-off plot, composites were computed at the response level using 0–1 min–max scaling across all 100 responses. The reliability composite was defined as the unweighted mean of scaled DISCERN total, EQIP total, JAMA total, and GQS (higher values indicate better quality). The readability composite was defined as the unweighted mean of scaled readability indices with directionality aligned so that higher values indicated easier readability: FRES retained its original direction (higher = easier), while ARI, GFI, FKGL, CL, and SMOG were inverted after scaling (1 – scaled value). Model-level composites were summarized as medians across each model's 20 responses.

For pairwise differences heatmap, cells display –log10 (p_holm), where p_holm is the Holm-adjusted *P*–value from the corresponding pairwise test; larger values indicate stronger evidence of a between-model difference. All figures and visualizations were produced using Flourish Studio (Flourish.studio). Additional descriptive statistics and item-level prevalence are provided in Supplementary Tables S7–S10.

### Reporting guideline

This study was reported in accordance with the CHART statement. The completed CHART checklist is provided in the Supplementary Table S5. Prompts are listed in Table 1, and the question-to-guideline mapping (including anchor PMIDs) is provided in Supplementary Table S1.

### Reporting and ethics considerations

This study evaluated outputs from publicly available AI systems using guideline-derived, non-patient prompts. No human participants were enrolled, and no identifiable private data were collected or analyzed.

## Results

### Patient-facing question set and analytic units

The final guideline-derived question set prompt set consisted of 20 single-intent, patient-facing IBD questions covering the care pathway (Table 1). These prompts generated 100 model–question responses across five LLMs (5 models × 20 questions), which served as the unit of analysis. No refusals, safety-policy non-answers, or otherwise non-responsive outputs occurred during data collection; refusal rates were 0% overall (0/100) and 0/20 for each model.

### Interrater agreement

Interrater agreement for the 100 model–question units was high for the prespecified primary outcomes (Table 2): DISCERN total, ICC(A,1) = 0.842; EQIP total, ICC(A,1) = 0.760; GQS, weighted κ = 0.812; and JAMA total, weighted κ = 0.936.

### Reliability and transparency-proxy performance

**Figure 1:**
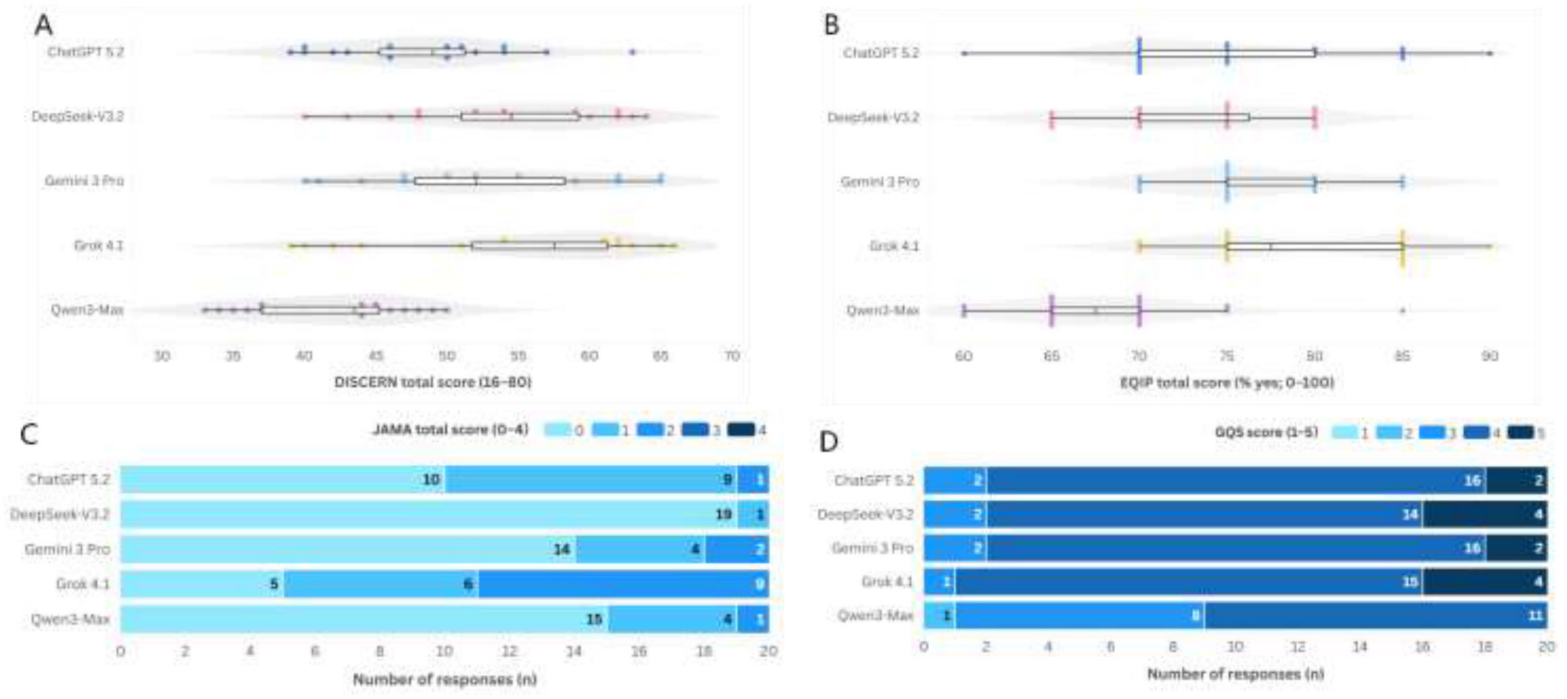
Distribution of reliability and transparency-proxy scores across 5 AI models in response to 20 IBD-related questions. **(A)** DISCERN total (16–80). **(B)** EQIP total (% yes; 0–100). **(C)** JAMA total (0–4). **(D)** GQS (1–5). Boxes denote the interquartile range (IQR) with the median as a horizontal line; whiskers indicate 1.5 × IQR; points represent individual question-level outputs (one point per response).

Model-level reliability metrics are summarized in Table 3, with response-level distributions shown in Figure 1. Overall, greater separation between models was observed for DISCERN and EQIP compared to GQS, while JAMA totals were consistently low across all models.

**Table 3:** Reliability scores across models (*n* = 20 questions per model).

Model	DISCERN total score (16–80)	EQIP total (% yes; 0–100)	JAMA total score (0–4)	GQS (1–5)
ChatGPT 5.2	48.45 ± 6.08 49.00 [45.25, 51.25]	75.00 ± 7.25 75.00 [70.00, 80.00]	0.55 ± 0.60 0.50 [0.00, 1.00]	4.00 ± 0.46 4.00 [4.00, 4.00]
DeepSeek-V3.2	54.45 ± 6.80 54.50 [51.00, 59.25]	73.00 ± 5.48 75.00 [70.00, 76.25]	0.05 ± 0.22 0.00 [0.00, 0.00]	4.10 ± 0.55 4.00 [4.00, 4.00]
Gemini 3 Pro	53.00 ± 7.43 52.00 [47.75, 58.25]	76.50 ± 4.89 75.00 [75.00, 80.00]	0.40 ± 0.68 0.00 [0.00, 1.00]	4.00 ± 0.46 4.00 [4.00, 4.00]
Grok 4.1	55.25 ± 8.29 57.50 [51.75, 61.25]	79.25 ± 6.34 77.50 [75.00, 85.00]	1.20 ± 0.83 1.00 [0.75, 2.00]	4.15 ± 0.49 4.00 [4.00, 4.00]
Qwen3-Max	41.85 ± 5.23 43.50 [37.00, 45.25]	68.00 ± 5.94 67.50 [65.00, 70.00]	0.30 ± 0.57 0.00 [0.00, 0.25]	3.50 ± 0.61 4.00 [3.00, 4.00]

Cells display the mean ± SD on the first line and the median [Q1, Q3] on the second line.

For DISCERN, median total scores ranged from 43.50 (Qwen3-Max) to 57.50 (Grok 4.1), with intermediate medians of 49.00 (ChatGPT 5.2), 52.00 (Gemini 3 Pro), and 54.50 (DeepSeek-V3.2) (Table 3). EQIP medians ranged from 67.50 (Qwen3-Max) to 77.50 (Grok 4.1). JAMA totals reflected limited transparency signals overall (median 0.00–1.00), with Grok 4.1 showing the highest central tendency (median 1.00 [0.75–2.00]). GQS medians were uniformly 4.00 across all models (Table 3; Figure 1). (As defined in the study, GQS is a 5-point scale ranging from 1 [very poor] to 5 [excellent].)

### Readability performance

**Figure 2:**
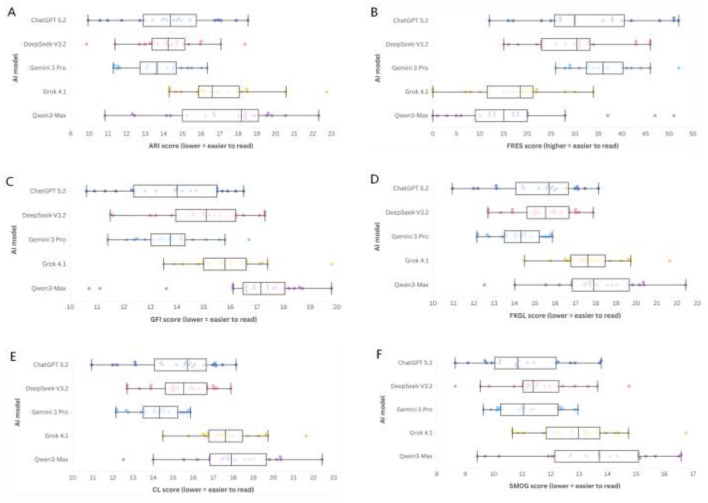
Distribution of readability scores across 5 AI models in response to 20 IBD-related questions. Box-and-whisker plots with overlaid individual data points compare readability across six indices: **(A)** ARI, **(B)** FRES, **(C)** GFI, **(D)** FKGL, **(E)** CL, and **(F)** SMOG. Lower values indicate easier readability for ARI/GFI/FKGL/CL/SMOG; higher values indicate easier readability for FRES. Reference thresholds are shown for sixth-grade readability (scores <6 for ARI/GFI/FKGL/CL/SMOG and FRES >80). Boxes denote the interquartile range (IQR) with the median as a horizontal line; whiskers indicate 1.5 × IQR; points represent individual question-level outputs (one point per response).

Readability metrics are summarized in Table 4 and displayed as response-level distributions in Figure 2. Across models, grade-level indices consistently exceeded sixth-grade targets, and FRES values were uniformly low compared to the conventional “easy” threshold.

**Table 4:** Readability metrics across models (*n* = 20 questions per model).

Model	ARI	FRES	GFI	FKGL	CL	SMOG
ChatGPT 5.2	14.30 ± 2.39 14.34 [12.90, 15.75]	32.95 ± 11.97 30.00 [25.75, 40.50]	13.79 ± 1.82 14.00 [12.38, 15.50]	12.53 ± 2.01 12.52 [11.07, 14.16]	15.18 ± 2.00 15.71 [14.07, 16.66]	11.06 ± 1.46 10.84 [10.04, 12.19]
DeepSeek-V3.2	14.25 ± 1.88 14.24 [13.37, 15.12]	29.75 ± 9.41 30.50 [23.00, 33.25]	14.96 ± 1.71 15.10 [13.95, 16.20]	12.97 ± 1.58 13.02 [12.23, 13.71]	15.41 ± 1.51 15.54 [14.62, 16.68]	11.58 ± 1.42 11.39 [11.03, 12.29]
Gemini 3 Pro	13.69 ± 1.58 13.64 [12.72, 14.68]	36.40 ± 6.57 36.00 [32.50, 40.25]	13.81 ± 1.31 13.75 [13.03, 14.30]	12.36 ± 1.31 12.19 [11.64, 13.63]	14.22 ± 1.18 14.32 [13.52, 15.23]	11.23 ± 1.06 11.05 [10.25, 12.26]
Grok 4.1	17.00 ± 2.10 16.61 [15.87, 18.05]	16.95 ± 8.94 18.50 [11.50, 21.25]	15.89 ± 1.41 15.80 [15.00, 16.60]	15.26 ± 1.82 15.04 [14.19, 16.25]	17.75 ± 1.56 17.61 [16.78, 18.46]	12.88 ± 1.55 12.98 [11.84, 13.73]
Qwen3-Max	17.08 ± 3.04 18.18 [15.01, 19.08]	17.40 ± 14.01 15.00 [9.00, 20.00]	16.65 ± 2.35 17.15 [16.48, 18.05]	15.58 ± 2.72 16.39 [14.75, 17.16]	17.98 ± 2.32 17.90 [16.84, 19.65]	13.47 ± 2.12 13.71 [12.14, 15.09]

Cells display the mean ± SD on the first line and the median [Q1, Q3] on the second line. For ARI, GFI, FKGL, CL, and SMOG, lower values indicate easier readability; for FRES, higher values indicate easier readability.

Gemini 3 Pro exhibited relatively easier readability on multiple grade-level indices (e.g., median ARI 13.64, median FKGL 12.19, median SMOG 11.05), while Grok 4.1 and Qwen3-Max demonstrated higher (more difficult) grade-level indices (e.g., median ARI 16.61 and 18.18, respectively) (Table 4; Figure 2). FRES medians ranged from 15.00 to 36.00 across models, all well below the 80 threshold.

### Overall between-model differences

Within-question paired testing revealed statistically significant between-model differences for all four reliability/transparency-proxy outcomes and all six readability indices (Friedman tests, *df* = 4; all Holm-adjusted *P* < 0.001 across the 10 outcomes) (Table 5). Effect sizes (Kendall's W) varied by outcome, ranging from 0.238 for GQS to 0.702 for CL, indicating the largest separation between models for selected readability indices and smaller separation for the single-item global quality rating (Table 5).

**Table 5:** Overall between-model differences (Friedman test; within-question paired, *n* = 20; *k* = 5).

Outcome	Friedman χ^2^	df	P	Kendall W	P (Holm, across outcomes)
DISCERN total score (16–80)	50.061	4	<0.001	0.626	<0.001
EQIP total (% yes; 0–100)	31.296	4	<0.001	0.391	<0.001
JAMA total score (0–4)	35.759	4	<0.001	0.447	<0.001
GQS (1–5)	19.028	4	<0.001	0.238	<0.001
ARI	26.400	4	<0.001	0.330	<0.001
FRES	50.589	4	<0.001	0.632	<0.001
GFI	37.648	4	<0.001	0.471	<0.001
FKGL	38.400	4	<0.001	0.480	<0.001
CL	56.120	4	<0.001	0.702	<0.001
SMOG	29.080	4	<0.001	0.363	<0.001

Holm adjustment in the last column is applied across the 10 outcomes in this table.

### Normalized profiles and composite trade-off

**Figure 3:**
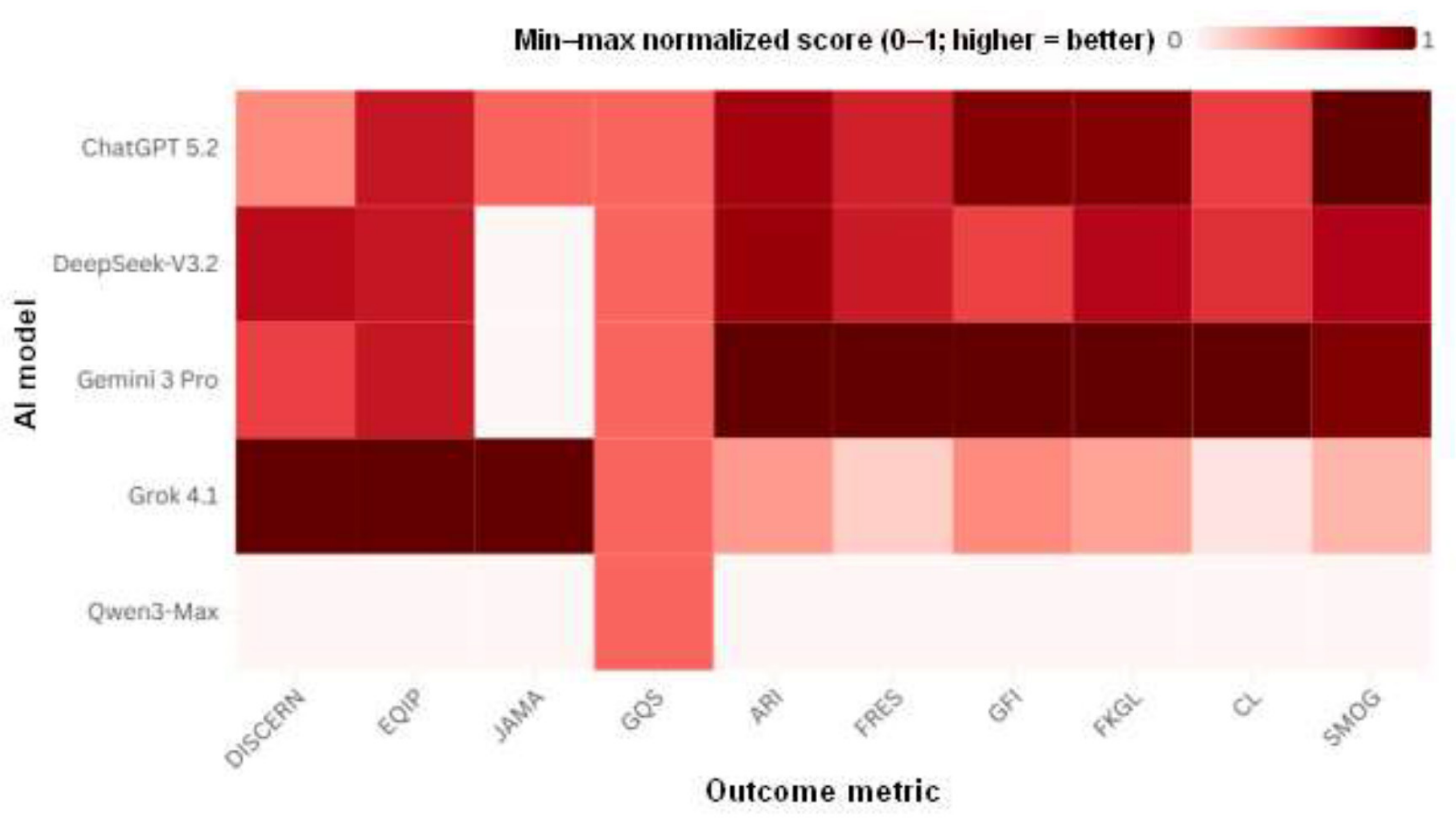
Min-max normalized heatmap of reliability and readability metrics across 5 AI models (*n* = 20 responses per model). Values are scaled to 0–1 within each metric across all responses (higher = better); darker shading indicates better normalized performance. For readability indices where lower values indicate easier readability (ARI/GFI/FKGL/CL/SMOG), directionality was aligned so higher values indicate easier readability; FRES retained its original direction (higher is easier).

**Figure 4:**
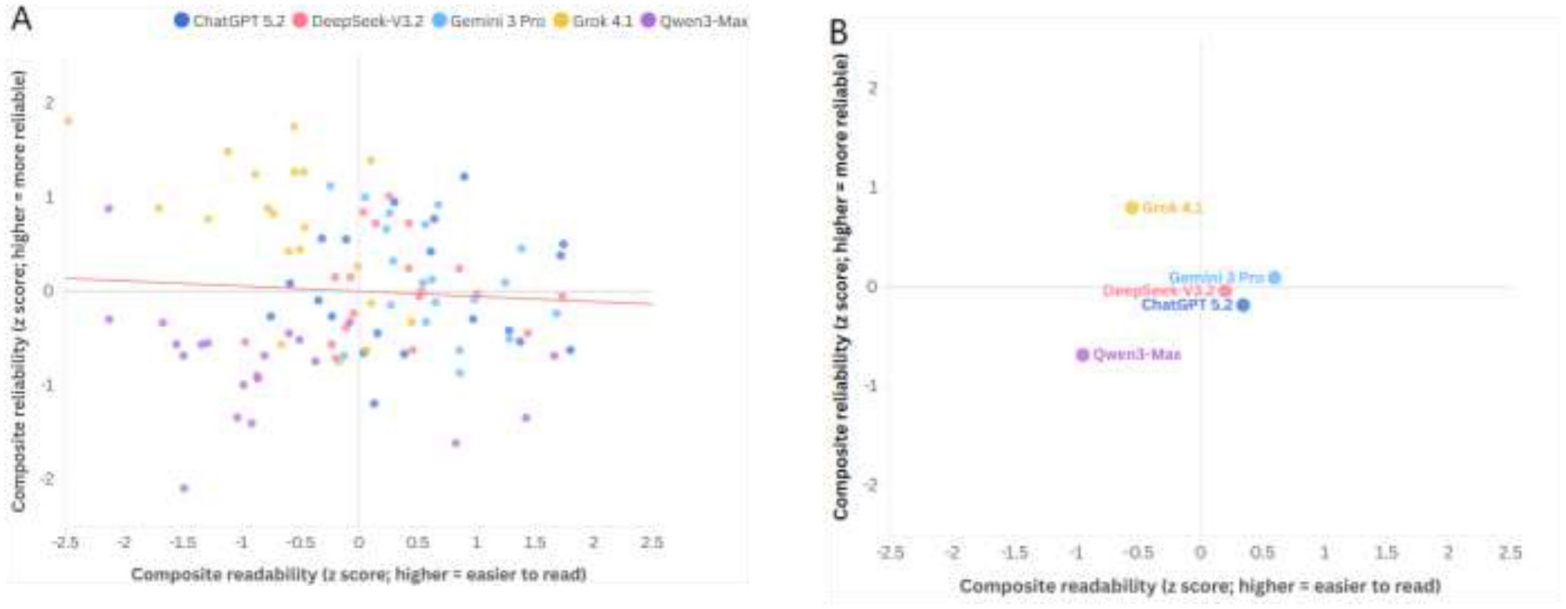
Trade-off between composite readability and composite reliability across models. **(A)** Response-level trade-off (*n* = 100). **(B)** Model-level medians. Composites were computed at the response level using 0–1 min-max scaling across all responses such that higher values indicate better performance. Reliability composite is the unweighted mean of scaled DISCERN total, EQIP total, JAMA total, and GQS. Readability composite is the unweighted mean of direction-aligned readability indices (FRES retained; ARI/GFI/FKGL/CL/SMOG inverted after scaling). The solid trend line in **(A)** represents the least-squares linear fit across all response-level points, summarizing the overall association between composite readability and composite reliability.

Figure 3 summarizes model performance patterns across reliability and readability metrics on a normalized scale. Figure 4 shows the relationship between composite readability and composite reliability at the response level (*n* = 100) and by model medians. The fitted trend line in Figure 4A summarizes the overall association across response-level points.

### Pairwise comparisons

**Figure 5:**
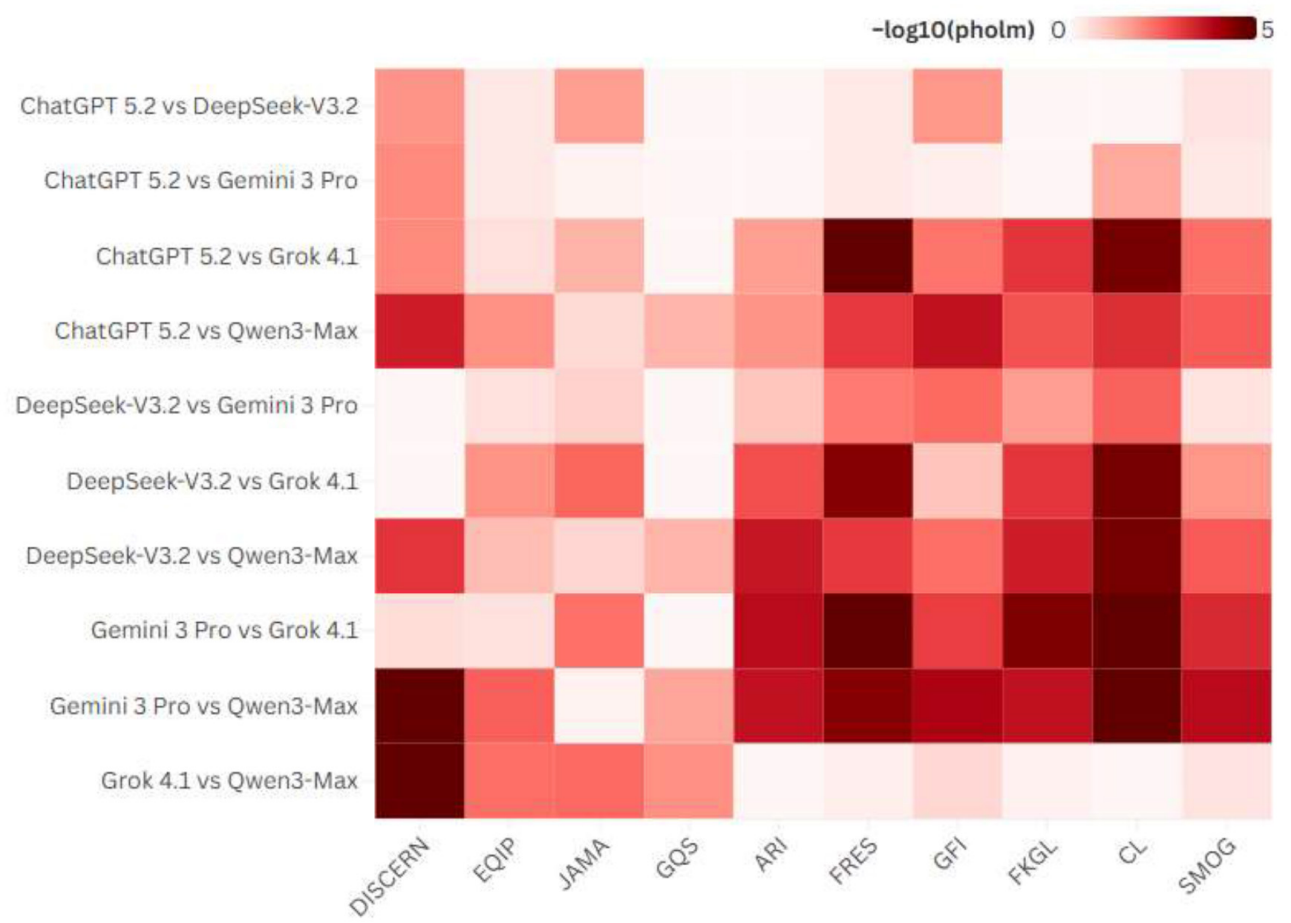
Pairwise differences in quality, reliability, and readability across large language models (Holm-adjusted). Heatmap shows pairwise comparisons between models (rows) across ten outcome metrics (columns). Each cell reports –log10(p_holm), where p_holm is the Holm-adjusted *P*-value from the corresponding two-sided Wilcoxon signed-rank test; larger values indicate stronger evidence of a between-model difference. Holm adjustment is applied within each outcome across the 10 pairwise comparisons. Raw (unadjusted) *P*-values for the primary outcomes are provided in Table 6.

Raw two-sided Wilcoxon signed-rank *P*-values for pairwise differences in the four primary outcomes (DISCERN, GQS, EQIP, and JAMA) are reported in Table 6. Figure 5 visualizes Holm-adjusted evidence strength for pairwise differences across all 10 outcomes (4 reliability/transparency-proxy outcomes and 6 readability indices) using –log10 (p_holm), where larger values indicate stronger evidence of a between-model difference after multiplicity control within each outcome.

**Table 6:** Pairwise comparison raw *P*-values (Wilcoxon signed-rank test; two-sided).

Comparison	DISCERN	GQS	EQIP	JAMA
ChatGPT 5.2 vs. DeepSeek-V3.2	0.0048	0.4795	0.2694	0.0039
ChatGPT 5.2 vs. Gemini 3 Pro	0.0024	1	0.4641	0.4054
ChatGPT 5.2 vs. Grok 4.1	0.0023	0.2568	0.0708	0.0097
ChatGPT 5.2 vs. Qwen3-Max	<0.0001	0.0075	0.0021	0.0956
DeepSeek-V3.2 vs. Gemini 3 Pro	0.5191	0.5271	0.0728	0.0384
DeepSeek-V3.2 vs. Grok 4.1	0.6215	0.7055	0.0025	0.0005
DeepSeek-V3.2 vs. Qwen3-Max	0.0001	0.008	0.014	0.0588
Gemini 3 Pro vs. Grok 4.1	0.1066	0.3657	0.1229	0.0008
Gemini 3 Pro vs. Qwen3-Max	<0.0001	0.0039	0.0004	0.4795
Grok 4.1 vs. Qwen3-Max	<0.0001	0.0016	0.0007	0.0006

Values are raw (unadjusted) P-values. Holm-adjusted P-values are provided in Supplementary Table S6.

## Discussion

In this guideline-derived question set, cross-sectional evaluation of 100 model–question responses generated by five publicly available LLMs in response to 20 patient-facing IBD questions spanning the care continuum (Table 1), three key findings stand out. First, models exhibited significant variability on multi-item instruments assessing informational quality and completeness (DISCERN and EQIP). Second, transparency signals—assessed *via* the JAMA benchmark criteria—were consistently lacking, indicating that default outputs rarely provide the necessary verifiability cues for patients to assess credibility, conflicts of interest, or currency. Third, readability was universally suboptimal across all models: grade-level indices far exceeded the recommended sixth-grade targets, and Flesch Reading Ease remained in the “difficult” range (Tables 3, 4; Figures 1–4). Notably, no evaluated model met the prespecified targets for patient-level readability or transparency under default settings. These findings suggest that while contemporary LLMs, in their default configurations, can generate fluent educational text, they do not reliably meet the standards required for patient-facing materials that need to be both auditable and accessible.

### Interpretation and reproducibility

A critical prerequisite for interpreting the observed between-model differences is measurement reproducibility. Interrater agreement for the prespecified primary outcomes was high (Table 2), with ICC(A,1) of 0.842 for DISCERN total, 0.760 for EQIP total, and weighted κ values of 0.812 for GQS and 0.936 for the JAMA benchmark total. This reliability does not establish construct validity but alleviates concerns that the observed differences reflect rater noise rather than systematic properties of the model outputs.

Between-model separation was most apparent for DISCERN and EQIP (Table 3; Figure 1). For DISCERN, median totals ranged from 43.5 (Qwen3-Max) to 57.5 (Grok 4.1), with intermediate medians of 49.0 (ChatGPT 5.2), 52.0 (Gemini 3 Pro), and 54.5 (DeepSeek-V3.2). For EQIP, medians ranged from 67.5 (Qwen3-Max) to 77.5 (Grok 4.1), with other models clustering around 75.0. These differences are clinically meaningful in IBD, where patient decisions rely on conditional logic (e.g., phenotype, severity, prior biologic exposure, infection-risk mitigation, monitoring cadence, and escalation strategies). Omitting qualifiers, timelines, or “what to do next” guidance can compromise decision quality, even when core concepts are directionally accurate.

In contrast, GQS medians were uniformly 4.0 across all models (Table 3), reflecting limited differentiation from this single-item gestalt measure and suggesting a ceiling effect driven by attributes where LLMs are broadly strong (e.g., fluency, organization, and confident tone). This pattern aligns with prior research showing that human preference ratings can diverge from decision-relevant quality: in one prominent evaluation, lay raters often preferred chatbot responses over physician responses, likely due to conversational completeness and tone rather than formal verifiability or guideline alignment (5, 44). Methodologically, our findings emphasize the importance of prioritizing multi-item instruments that operationalize decision-critical elements (e.g., DISCERN/EQIP) rather than relying on global impressions alone when evaluating patient-facing LLM outputs.

### Transparency and verifiability

Transparency (JAMA benchmark) was low across models, with medians typically ranging between 0 and 1 out of 4 (Table 3; Figure 1). Item-level JAMA signals were sparse under default settings: authorship and disclosure were absent in the response text (0/100 each), whereas attribution cues were observed in 37/100 (37%) and currency cues in 13/100 (13%). These findings indicate that low JAMA totals primarily reflect limited default verifiability scaffolding in conversational outputs rather than implying poor factual accuracy. Even the highest-performing model on this metric, Grok 4.1, had a median of 1.0 [0.75–2.0], while several models had medians at or near 0. These results suggest that default LLM outputs rarely provide authorship, attribution, disclosure, or currency cues necessary for users to evaluate credibility, contextualize uncertainty, or recognize time sensitivity. Low transparency does not necessarily imply incorrect content; rather, it indicates that patients are not provided with the scaffolding required to appropriately weigh recommendations or reconcile conflicting advice—an especially critical gap in IBD-related topics such as immunosuppression, vaccination, infection screening, and surveillance intervals.

A critical distinction is that simply “adding references” does not equate to verifiability. Numerous studies show that LLM-generated citations can be fabricated or inaccurate (45–47). Walters and Wilder demonstrated substantial fabrication and errors in bibliographic citations generated by ChatGPT, emphasizing that seemingly plausible reference lists can include nonexistent works or incorrect metadata (48). Similarly, Chelli et al. found notable hallucination and reference inaccuracies when LLMs attempted systematic review-style reference retrieval (14). These findings highlight the need for a practical distinction in patient-facing systems: the presence of citations does not guarantee citation accuracy. For provenance to be meaningful, references must be (1) real, (2) correctly identified (e.g., DOI/PMID matches title and journal), and (3) linked to the specific claims they are meant to support.

This issue is particularly relevant for IBD, where guideline recommendations evolve and are often conditional. In an ECCO-guideline–linked evaluation, ChatGPT was frequently accurate in providing IBD patient information, though some deviations occurred, and certain areas required clearer, more actionable guidance (49). The alignment between these findings and our results suggests a pragmatic conclusion: even when LLMs are “often correct,” default outputs do not reliably equip patients to assess credibility, detect outdated advice, or understand the conditional nature of recommendations.

From an implementation perspective, transparency should be treated as an essential output specification, not a stylistic optionality (50). The CHART reporting guideline highlights the importance of transparent description and interpretability for chatbot health advice studies and, by extension, offers a useful conceptual framework for deployment expectations (51). For patient-facing IBD applications, structured fields could directly operationalize multiple transparency elements without relying on users to request them: (1) “Sources used” (guideline titles plus PMIDs/DOIs), (2) “As of” date (currency), (3) Authorship/disclosure (system identity, limitations, conflicts), and (4) “What this answer is (and is not)” (educational summary vs. individualized medical advice). When citations are provided, systems should prioritize machine-verifiable identifiers (PMID/DOI) and incorporate lightweight validation (e.g., flagging nonexistent identifiers or title–identifier mismatches). This “verify-by-construction” approach addresses the failure modes documented in citation hallucination studies and aligns with the low JAMA benchmark totals observed in this study (45).

### Readability and accessibility

Across models, readability indices consistently exceeded sixth-grade targets, and Flesch Reading Ease scores remained low (Table 4; Figure 2). For instance, median FKGL ranged from 12.2 (Gemini 3 Pro) to 16.4 (Qwen3-Max), and median ARI ranged from 13.6 to 18.2. Median FRES values ranged from 15.0 (Qwen3-Max) to 36.0 (Gemini 3 Pro), all of which are considered difficult for broad patient audiences. Although relative differences exist, the overall readability level indicates that default, one-shot responses are linguistically complex and likely to pose challenges for users with limited health literacy or those seeking information during high-stress situations (e.g., flare symptoms, fever while on immunosuppression) (52).

Encouragingly, external evidence suggests that readability can be improved, typically through constrained rewriting rather than unconstrained generation. Will and colleagues demonstrated that prompting LLMs to simplify online patient education materials improved multiple readability metrics and reduced word counts, though achieving a strict sixth-grade target was not consistently achieved across materials and models (53). In another evaluation of pediatric orthopedic education materials, currently available LLMs were moderately effective at enhancing readability in both English and Spanish, again indicating that readability improvements are possible, but not guaranteed to meet stringent targets (54). These studies support a practical interpretation of our results: readability issues are not necessarily a fixed model limitation, but rather a predictable consequence of unconstrained generation without explicit readability goals and post-generation safeguards.

Accordingly, readability control should be prioritized as a first-order objective for patient-facing IBD systems. A pragmatic design approach is layered communication: a concise, plain-language summary (explicitly constrained to a sixth-grade target) focusing on actionable steps (e.g., red flags, monitoring, questions to ask), paired with an expandable “details” section containing conditional logic (e.g., phenotype/severity, prior biologic exposure, infection risk mitigation) and guideline-linked provenance. This structure maintains decision relevance while reducing the cognitive burden of reading a response at a higher grade level.

### Performance heterogeneity

Overall, between-model differences were statistically significant across outcomes (Table 5), with Kendall W indicating large effects for several readability metrics (e.g., CL W = 0.702, FRES W = 0.632) and for DISCERN (*W* = 0.626). These findings suggest that “model performance” is multidimensional; models that perform well on one axis may not do so on another. The normalized profiles (Figure 3) and composite plot (Figure 4) capture this heterogeneity, revealing an inverse trend between composite reliability and composite readability. This pattern should be interpreted descriptively: the study is not designed to infer causality, and it does not suggest that improving accessibility must compromise informational quality. In fact, the readability-rewriting literature indicates that accessibility can be improved *post hoc*, although there is a non-zero risk of meaning distortion or inaccuracies, which highlights the need for review and validation in high-risk topics (53, 54).

### Implications for practice and health systems

These results suggest several practical actions. First, when patients present LLM-generated content during visits, clinicians may focus on provenance and conditionality—whether the response cites guideline sources, includes an “as of” date, distinguishes general information from individualized care, and clearly defines when urgent evaluation is necessary. Second, health systems considering patient-facing LLM tools for IBD should implement safeguards directly addressing the failure modes observed here: (1) structured transparency fields (sources/currency/disclosure), (2) readability-constrained opening summaries, (3) standardized red-flag triage language, and (4) citation-validation and evidence anchoring.

More broadly, safety research highlights that medical LLMs can be manipulated or produce plausible yet false outputs under certain conditions, including through training-data poisoning or “helpfulness” behaviors that reinforce user misconceptions (sycophancy) (55). These risks emphasize that deployment should not rely on surface fluency as a proxy for safety. Instead, systems should incorporate auditable retrieval, validation, and monitoring mechanisms that prioritize honesty, uncertainty communication, and verifiability.

### Strengths, limitations, and future directions

Strengths of this study include a standardized, guideline-derived question set covering major decision points across the IBD care pathway (Table 1); the use of established instruments with high interrater agreement (Table 2); and a multiplicity-controlled analytic framework (Tables 5, 6). The use of multiple readability indices mitigates overreliance on any single formula and supports robust directional conclusions (Table 4).

However, this study has several limitations. First, outputs were generated within a defined time window using publicly accessible interfaces, and model updates or interface-level features may affect performance over time. Specifically, queries were run during a prespecified window with public web interfaces in default configurations (incognito/private browsing, new session per question to minimize carryover, no API or retrieval augmentation). These implementation details may limit generalizability as vendors continue to iterate on models and user-facing features. Second, while prompts were standardized to maximize cross-model comparability, real-world users differ in language, context, and iterative follow-up; thus, performance in multi-turn dialogues may vary. Third, because large language model outputs are stochastic, we evaluated a single response per model per question and did not quantify within-model variability across repeated generations under identical conditions. Accordingly, the observed between-model differences should be interpreted as differences among single sampled outputs rather than estimates of average model performance. Fourth, readability formulas are proxies for comprehensibility and do not capture factors such as medical numeracy, layout and formatting effects, or the influence of visual aids and other interface features on understanding. Fifth, although our instruments assess perceived quality, completeness, transparency proxies, and readability, they do not provide a question-specific gold-standard verification of factual correctness, nor do they evaluate downstream effects on patient knowledge, decision-making, or clinical outcomes. Sixth, the JAMA benchmark was originally developed for static health websites; some criteria (e.g., named authorship, disclosures, and update dates) may be structurally less likely to appear in default conversational outputs unless explicitly requested or provided outside the response text. Accordingly, JAMA scores should be interpreted as a minimum transparency level of the response text and may underestimate platform-level transparency features. Accordingly, JAMA scores should be interpreted as the minimum transparency conveyed by the response text and may underestimate transparency available at the interface level. Overall, these findings should be interpreted as an assessment of default output characteristics rather than conclusive evidence of clinical safety.

Future work should focus on interventions that directly address observed weaknesses: (1) transparency enforcement (e.g., mandatory sources/currency fields with auditable identifiers and automated validation), (2) readability control (e.g., automatic rewriting to defined thresholds with meaning-preservation checks and escalation for high-risk topics), and (3) layered output templates that preserve conditional logic while providing accessible front-end guidance. Longitudinal monitoring across model versions will be crucial, given the rapid pace of model iteration. Finally, patient-centered studies measuring comprehension, decision quality, and appropriate help-seeking behavior would provide stronger evidence than readability proxies alone.

## Conclusion

In conclusion, across guideline-derived IBD patient questions, publicly available LLMs produced outputs with varying levels of informational quality and completeness but were consistently characterized by weak transparency signals and limited readability. Under default settings, none met the prespecified thresholds for transparency or patient-level readability. These deficits appear addressable but are unlikely to improve reliably unless verifiability and accessibility are treated as explicit output requirements. For patient-facing IBD applications, structured provenance fields, readability-constrained summaries, layered presentation, and validation safeguards may be essential to align LLM-generated content with the standards expected of high-quality patient information.

## Data Availability

The raw data supporting the conclusions of this article will be made available by the authors, without undue reservation.
