# Supplementary material for: Benchmarking public large language model responses to patient-facing inflammatory bowel disease questions: informational quality, transparency proxies, and readability
Source: Front Public Health. 2026 Apr 10;14:1810358. doi: 10.3389/fpubh.2026.1810358 (PMC13106179; doi:10.3389/fpubh.2026.1810358)
Supplement: Supplementary file 1 [file Table_1.docx]

**Supplementary Materials**

Supplementary Methods. Statistical details for revised tables/figures

Descriptive statistics are reported as mean ± SD and median [Q1, Q3] per model (n=20 question-level responses per model). Overall between-model differences were tested with the Friedman test (within-question paired; k=5 models), with Kendall’s W reported as an effect size. For Table 5, Holm adjustment was applied across the 10 outcomes. For pairwise comparisons, Wilcoxon signed-rank tests (two-sided; zero differences dropped) were used; Holm adjustment was applied within each outcome across the 10 pairwise model comparisons. Effect size r is the rank-biserial correlation computed from signed-rank sums (positive indicates the first-listed model scores higher).

Supplementary Methods. Composite readability and reliability (for Figure 4)

Composites were computed at the response level using 0–1 min–max scaling across all 100 responses. Reliability composite is the unweighted mean of scaled DISCERN total, EQIP total, JAMA total, and GQS (higher is better). Readability composite is the unweighted mean of scaled readability indices with directionality aligned so higher is easier to read: FRES retained its original direction (higher is easier), whereas ARI/GFI/FKGL/CL/SMOG were inverted after scaling (1−scaled value). Model-level composites are reported as medians across each model’s 20 responses.

Supplementary Table S1. Question-to-Guideline Mapping

| Q# | Patient-facing question  (English) | Domain | China guideline anchor (CN) | International guideline anchor (INT) |
| --- | --- | --- | --- | --- |
| 1 | Difference between UC and CD | Basics/phenotype | CN3 | INT5/INT6 |
| 2 | Tests used to diagnose IBD | Diagnosis | CN3, CN1/CN2 | INT5/INT6 |
| 3 | Active vs remission | Activity assessment | CN1, CN2 | INT5 |
| 4 | Monitoring over time | Monitoring | CN1, CN2 | INT5 |
| 5 | Symptoms needing urgent care | Safety/triage | CN1, CN2 | INT5, INT2 |
| 6 | Severe UC flare in hospital | Acute severe UC | CN1 | INT2 |
| 7 | Surgery recommended for Crohn’s disease | Surgery (CD) | CN2 | INT4 |
| 8 | First-line mild–moderate UC | Treatment (UC) | CN1 | INT1 |
| 9 | First-line mild–moderate CD | Treatment (CD) | CN2 | INT3 |
| 10 | When steroids are used | Steroids (induction) | CN1, CN2 | INT1, INT3 |
| 11 | Why no long-term steroids | Steroid stewardship | CN1, CN2 | INT12 |
| 12 | When to start biologics/small molecules | Escalation | CN1, CN2 | INT1, INT3 |
| 13 | Options if biologic stops working | Loss of response | CN1, CN2 | INT1, INT3 |
| 14 | Infection screening before immunosuppression | Infection prevention | CN4 | INT6 |
| 15 | Vaccines on immunosuppression | Vaccination | CN4 | INT6, INT12 |
| 16 | Fever while on immunosuppressants | Infection management | CN4 | INT6, INT12 |
| 17 | CRC surveillance colonoscopy interval | Cancer surveillance | CN1, CN2, CN3 | INT11, INT9 |
| 18 | Pregnancy & breastfeeding meds | Pregnancy | CN3 | INT7 |
| 19 | Nutrition & micronutrient deficiencies | Nutrition | CN3 | INT10 |
| 20 | Treating iron-deficiency anemia | Anemia | CN3 | INT8 |
| Key Guideline Anchors  China (CN)  CN1: 2023 UC Clinical Practice Guideline (PMID: 38955435)  CN2: 2023 CD Clinical Practice Guideline (PMID: 38955665)  CN3: 2018 Chinese Consensus on IBD (PMID: 33905603)  CN4: Consensus on Infections in IBD (PMID: 29330905)  International (INT)  INT1: ECCO UC Medical Treatment Guidelines (PMID: 34635919)  INT2: ECCO ASUC Management Guidelines (PMID: 34635910)  INT3: ECCO CD Medical Treatment Guidelines (PMID: 38877997)  INT4: ECCO CD Surgical Treatment Guidelines (PMID: 38878002)  INT5: ECCO Diagnostics & Monitoring Guidelines (PMID: 40741688)  INT6: ECCO Infections & Vaccination Guidelines (PMID: 33730753)  INT7: ECCO Pregnancy & Lactation Guidelines (PMID: 36005814)  INT8: ECCO Extraintestinal Manifestations Guidelines (PMID: 37351850)  INT9: ECCO Colorectal Cancer Surveillance Guidelines (PMID: 40306978)  INT10: ESPEN Clinical Nutrition Guidelines (PMID: 36739756)  INT11: BSG Colorectal Surveillance Guidelines (PMID: 40701559)  INT12: ACG Preventive Care Guidelines (PMID: 40701559) | | | | |

Supplementary Table S2. EQIP-20 checklist and operationalization for rating short-form LLM responses

EQIP (Ensuring Quality Information for Patients) was applied at the response level (one model answer to one patient question). Each of the 20 items was rated as Yes (1) or No (0) using the prespecified decision rules below. The EQIP total score was calculated as (%Yes × 100) = (sum of Yes / 20) × 100. No items were omitted and no “Not applicable (N/A)” category was used; if an item was not explicitly satisfied in a given response, it was scored as No. Where an item could be considered context-dependent (e.g., action guidance), raters judged whether the response provided an appropriate equivalent for that question type.

| Item | EQIP item (short label) | Operational definition (score Yes if…) |
| --- | --- | --- |
| 1 | Source/origin stated | The response identifies a credible source type (e.g., guideline, society, hospital/clinic, peer-reviewed evidence) or clearly states that it is general educational information and recommends consulting a clinician/guideline for personalized care. |
| 2 | References provided | At least one verifiable reference cue is provided (e.g., guideline title/year, society guideline, PMID/DOI, named organization document, or a clearly identifiable citation). |
| 3 | Adequate background/context | Defines the topic and provides sufficient context (definitions, who it applies to, key concepts) before recommendations. |
| 4 | Accurate and unambiguous | Statements are clinically coherent and avoid internal contradictions; key recommendations are qualified where needed (e.g., ‘depends on severity/phenotype’) rather than presented as absolute when conditional. |
| 5 | Evidence-based/scientific | Recommendations are framed as evidence-based (e.g., aligns with guideline logic; avoids unproven claims; clearly distinguishes evidence-supported options from speculation). |
| 6 | Currency addressed | Includes an explicit ‘as of’ date/year, acknowledges that recommendations can change over time, or references a dated guideline/consensus (year stated). |
| 7 | Plain language | Uses patient-friendly wording and defines unavoidable medical terms in-line. |
| 8 | Helpful visuals/format aids | Uses an easy-to-follow table, stepwise algorithm, checklist, or other structured formatting that functions as a ‘visual aid’ in text (e.g., markdown table). |
| 9 | Key points easy to find | Highlights the most important content via headings, bullets, summary box, or an opening ‘key points’ section. |
| 10 | Clear structure | Logical organization (e.g., overview → key points → details → next steps), with coherent sectioning. |
| 11 | Practical usefulness | Provides actionable, applicable information for a patient (what to do/ask/monitor), not purely descriptive. |
| 12 | Bias/neutrality | Presents options fairly and avoids promotional tone, product/brand pushing, or one-sided framing. |
| 13 | Clear action guidance | Gives concrete guidance appropriate to the question (e.g., ‘when to seek urgent care’, ‘what to discuss with your doctor’, ‘typical monitoring’). |
| 14 | Privacy respected | Does not request identifiable personal information; if asking for context, uses non-identifying prompts (e.g., general symptoms) and includes a privacy caveat when relevant. |
| 15 | Minimizes jargon | Avoids unnecessary technical terms; when used, explains them in plain language. |
| 16 | Balanced perspective | Discusses benefits/risks/alternatives or uncertainty where relevant, rather than only benefits. |
| 17 | Engaging/presented well | Readable presentation that encourages continued reading (short paragraphs, bullets, headings; avoids wall-of-text). |
| 18 | Cultural/language needs considered | Acknowledges that recommendations/resources may differ by region and suggests seeking local guideline/clinician advice or provides culturally/regionally neutral phrasing. |
| 19 | Supports informed decisions | Explicitly supports decision-making (e.g., clarifies choices, tradeoffs, questions to ask, red flags, and decision criteria). |
| 20 | Clear conclusion/summary | Ends with a succinct conclusion or summary that reiterates key takeaways and next steps. |

Notes:

1) Item 8 was scored Yes when a response included a text-based table/checklist/algorithm (e.g., markdown table) that materially improved navigability; simple bullet lists alone did not qualify unless they functioned as a checklist with clear categories.

2) Item 6 was scored Yes only when the response explicitly addressed currency (year/date, ‘as of’ statement, or dated guideline reference). Generic statements such as ‘evidence suggests’ without any temporal cue were scored No.

3) Item 14 was scored Yes by default unless the response requested personally identifying details or encouraged sharing sensitive information.

4) Because EQIP was applied consistently across all 20 questions and all 100 responses, any consistently absent elements (e.g., lack of explicit references) affected absolute scores but did not introduce differential bias between models.

## Supplementary Table S3. Item-level operationalization of DISCERN for patient-facing IBD prompts (treatment and non-treatment questions)

Scoring anchors (apply to all items): 1 = not addressed/unclear; 3 = partially addressed (limited detail, incomplete qualifiers); 5 = explicit, correct, and sufficiently complete for the prompt.

NA handling: No items were considered “not applicable”; unaddressed items were scored 1.

| DISCERN item | Domain | Operational definition in this study (LLM response to a single prompt) | Application to non-treatment prompts (examples of “management-choice” analogs) |
| --- | --- | --- | --- |
| 1 | Reliability | States a clear aim/scope relevant to the prompt (what the answer will cover; educational intent). | Same (e.g., clarifies it will explain tests, monitoring and when to seek care). |
| 2 | Reliability | Achieves the stated aim: directly answers the prompt with adequate completeness. | Same. |
| 3 | Reliability | Relevance: information is on-topic and applicable to IBD. | Same. |
| 4 | Reliability | Sources: identifies information sources (guidelines/organizations) or provides verifiable references/authority names. | Same (higher if specific; lower if vague). |
| 5 | Reliability | Currency: indicates guideline year/“as of” timing or notes recommendations may change. | Same. |
| 6 | Reliability | Balanced/unbiased: avoids overstatement; notes pros/cons or uncertainty when appropriate. | Same. |
| 7 | Reliability | Additional support: suggests reputable resources or appropriate clinician follow-up (when indicated). | Same (e.g., contact IBD team; urgent care for red flags). |
| 8 | Reliability | Uncertainty/individualization: acknowledges variation by phenotype, severity, comorbidities, or clinician judgment. | Same. |
| 9 | Management choices (DISCERN Section 2) | Explains how each option works (mechanism/rationale). | Rationale for tests/monitoring/triage actions (e.g., why fecal calprotectin/CRP; why colonoscopy surveillance; why urgent evaluation). |
| 10 | Management choices | Describes benefits of each option. | Benefits of diagnostic/monitoring/preventive actions (earlier detection, relapse control, complication prevention). |
| 11 | Management choices | Describes risks/downsides of each option. | Risks/burdens/limitations of tests/monitoring/prevention (procedure risks, false positives, resource burden, delays). |
| 12 | Management choices | Describes what happens with no treatment. | “No action/no evaluation/no monitoring” analog (delayed diagnosis, missed flare, higher complication risk if red flags ignored). |
| 13 | Management choices | Describes impact on overall quality of life. | Impact of management pathways on daily life (symptoms, functioning, work/school, anxiety, diet/activities). |
| 14 | Management choices | Presents more than one choice or notes that decisions depend on context. | Alternatives/paths in evaluation and monitoring (different tests/intervals/settings); notes individualized decision factors. |
| 15 | Management choices | Supports shared decision-making: encourages discussion; lists decision factors; suggests questions to ask. | Same (emphasizes individualized planning with clinician; decision factors and trade-offs). |
| 16 | Overall | Overall quality rating based on Items 1–15 for the given prompt. | Same. |

Supplementary Table S4. GQS scoring rubric (anchor definitions) for rating short-form LLM responses

| Score | Anchor definition |
| --- | --- |
| 1 | Very poor: Inaccurate, misleading, not helpful |
| 2 | Poor: Limited accuracy, important gaps |
| 3 | Fair: Mixed quality; some useful info |
| 4 | Good: Mostly accurate, useful and organized |
| 5 | Excellent: High-quality, comprehensive, helpful to patients |

Supplementary Table S5. CHART Checklist

| **Heading** | **No** | **Chart checklist item** | **Page No** |
| --- | --- | --- | --- |
| **Title and abstract** |  |  |  |
| Title | 1a | State that the study is assessing one or more generative AI-driven chatbots for clinical evidence or health advice. | 1 |
| Abstract/summary | 1b | Apply a structured format, if applicable. | 2 |
| **Introduction** |  |  |  |
| Background | 2a | State the scientific background, rationale, and healthcare context for evaluating the generative AI-driven chatbot(s), referencing relevant literature when applicable. | 4 |
|  | 2b | State the aims and research questions including the target audience, intervention, comparator(s), and outcome(s). | 4 |
| **Methods** |  |  |  |
| Model identifiers | 3a | State the name and version identifier(s) of the generative AI model(s) and chatbot(s) under evaluation, as well as their date of release or last update. | 7-8 |
|  | 3b | State whether the generative AI model(s) and chatbot(s) are open-source or closed-source/proprietary. | 8 |
| Model details | 4a | State whether the generative AI model was a base model or a novel base model, tuned model, or fine-tuned model. | 8 |
|  | 4b | If a base model is used, cite its development in sufficient detail to identify the model. | 8 |
| Prompt engineering | 5a | Describe the evolution of study prompt development. | 6 |
|  | 5ai | Describe the sources of prompts. | 6 |
|  | 5aii | State the number and characteristics of the individual(s) involved in prompt engineering. | 7 |
|  | 5aiii | Provide details of any patient and public involvement during prompt engineering. | Not reported |
| Query strategy | 6a | State route of access to generative AI model. | 8 |
|  | 6b | State the date(s) and location(s) of queries for the generative AI-driven chatbot(s) including the day, month, and year as well as city and country. | 8 |
|  | 6c | Describe whether prompts were input into separate chat session(s). | 8 |
|  | 6d | Provide all generative AI-driven chatbot output/responses | Not provided |
| Performance evaluation | 7a | Define the ground truth or reference standard used to define successful generative AI-driven chatbot performance. | 9 |
|  | 7b | Describe the process undertaken for generative AI-driven chatbot performance evaluation. | 9 |
|  | 7bii | State the number and characteristics of team members involved in performance evaluation. | 9 |
|  | 7biii | Provide details of any patients and public involvement during the evaluation process. | Not reported |
| Sample size | 8 | Report how the sample size was determined. | 6 |
| Data analysis | 9a | Describe statistical analysis methods, including any evaluation of reproducibility of generative AI-driven chatbot responses. | 7 |
|  | 9ai | Report the measures used for performance evaluation. | 8 |
| **Results** |  |  |  |
|  | 10a | Report the performance evaluation undertaken including the alignment between generative AI-driven chatbot output and ground truth or reference standard using quantitative or mixed methods approaches as applicable. | 13 |
|  | 10b | For responses deviating from the ground truth or reference standard, state the nature of the difference(s). | 13 |
|  | 10c | Report the evaluation for potentially harmful, biased, or misleading responses. | Not reported |
| Discussion |  |  |  |
|  | 11a | Interpret study findings in the context of relevant evidence. | 16-18 |
|  | 11b | Describe the strengths and limitations of the study. | 21-22 |
|  | 11c | Describe the potential implications for practice, education, policy, regulation, and research. | 21 |
| Open science |  |  |  |
| Disclosures | 12a | Report any relevant conflicts of interest for all authors. | 24 |
| Funding | 12b | Report sources of funding and their role in the conduct and reporting of the study. | 24 |
| Ethics | 12c | Describe the processes undertaken for ethical approval. | 13 |
|  | 12ci | State whether permission/licensing was obtained for the use of original, copyrighted data. | Not applicable |
| Protocol | 12d | Provide a study protocol. | Not provided |
| Data availability | 12e | State where study data, code repository, and model parameters can be accessed. | Not provided |

Supplementary Table S6. Pairwise comparisons (Wilcoxon signed-rank) with raw P, Holm-adjusted P, and effect size r

| Outcome | Comparison | p_raw | p_holm_within_outcome | effect_r |
| --- | --- | --- | --- | --- |
| DISCERN total score (16–80) | ChatGPT 5.2 vs DeepSeek-V3.2 | 0.0047838511 | 0.019135405 | -0.736842 |
| DISCERN total score (16–80) | ChatGPT 5.2 vs Gemini 3 Pro | 0.0023620409 | 0.013950348 | -0.812865 |
| DISCERN total score (16–80) | ChatGPT 5.2 vs Grok 4.1 | 0.002325058 | 0.013950348 | -0.742857 |
| DISCERN total score (16–80) | ChatGPT 5.2 vs Qwen3-Max | 4.7683716e-05 | 0.00038146973 | 0.928571 |
| DISCERN total score (16–80) | DeepSeek-V3.2 vs Gemini 3 Pro | 0.5191486 | 1 | 0.168421 |
| DISCERN total score (16–80) | DeepSeek-V3.2 vs Grok 4.1 | 0.62151337 | 1 | -0.138095 |
| DISCERN total score (16–80) | DeepSeek-V3.2 vs Qwen3-Max | 0.00012713769 | 0.00088996381 | 1.000000 |
| DISCERN total score (16–80) | Gemini 3 Pro vs Grok 4.1 | 0.10657173 | 0.31971519 | -0.444444 |
| DISCERN total score (16–80) | Gemini 3 Pro vs Qwen3-Max | 1.9073486e-06 | 1.9073486e-05 | 1.000000 |
| DISCERN total score (16–80) | Grok 4.1 vs Qwen3-Max | 1.9073486e-06 | 1.9073486e-05 | 1.000000 |
| EQIP total (% yes; 0–100) | ChatGPT 5.2 vs DeepSeek-V3.2 | 0.26944023 | 0.53888046 | 0.316667 |
| EQIP total (% yes; 0–100) | ChatGPT 5.2 vs Gemini 3 Pro | 0.46410871 | 0.53888046 | -0.219048 |
| EQIP total (% yes; 0–100) | ChatGPT 5.2 vs Grok 4.1 | 0.07075893 | 0.35379465 | -0.542857 |
| EQIP total (% yes; 0–100) | ChatGPT 5.2 vs Qwen3-Max | 0.0020942383 | 0.016753906 | 0.836601 |
| EQIP total (% yes; 0–100) | DeepSeek-V3.2 vs Gemini 3 Pro | 0.072828879 | 0.35379465 | -0.516667 |
| EQIP total (% yes; 0–100) | DeepSeek-V3.2 vs Grok 4.1 | 0.0025273467 | 0.017691427 | -0.845588 |
| EQIP total (% yes; 0–100) | DeepSeek-V3.2 vs Qwen3-Max | 0.013979313 | 0.083875878 | 0.733333 |
| EQIP total (% yes; 0–100) | Gemini 3 Pro vs Grok 4.1 | 0.12288454 | 0.36865363 | -0.457143 |
| EQIP total (% yes; 0–100) | Gemini 3 Pro vs Qwen3-Max | 0.00037533216 | 0.0037533216 | 1.000000 |
| EQIP total (% yes; 0–100) | Grok 4.1 vs Qwen3-Max | 0.00065754878 | 0.005917939 | 0.934641 |
| JAMA total score (0–4) | ChatGPT 5.2 vs DeepSeek-V3.2 | 0.0038924171 | 0.02724692 | 1.000000 |
| JAMA total score (0–4) | ChatGPT 5.2 vs Gemini 3 Pro | 0.40538056 | 0.81076111 | 0.272727 |
| JAMA total score (0–4) | ChatGPT 5.2 vs Grok 4.1 | 0.0097190638 | 0.058314383 | -0.780220 |
| JAMA total score (0–4) | ChatGPT 5.2 vs Qwen3-Max | 0.095580705 | 0.28674211 | 0.555556 |
| JAMA total score (0–4) | DeepSeek-V3.2 vs Gemini 3 Pro | 0.03843393 | 0.19216965 | -1.000000 |
| JAMA total score (0–4) | DeepSeek-V3.2 vs Grok 4.1 | 0.00045109535 | 0.0045109535 | -1.000000 |
| JAMA total score (0–4) | DeepSeek-V3.2 vs Qwen3-Max | 0.058781721 | 0.23512689 | -1.000000 |
| JAMA total score (0–4) | Gemini 3 Pro vs Grok 4.1 | 0.00078585946 | 0.0062868757 | -1.000000 |
| JAMA total score (0–4) | Gemini 3 Pro vs Qwen3-Max | 0.47950012 | 0.81076111 | 0.333333 |
| JAMA total score (0–4) | Grok 4.1 vs Qwen3-Max | 0.00056533394 | 0.0050880054 | 1.000000 |
| GQS (0–5) | ChatGPT 5.2 vs DeepSeek-V3.2 | 0.47950012 | 1 | -0.250000 |
| GQS (0–5) | ChatGPT 5.2 vs Gemini 3 Pro | 1 | 1 | 0.000000 |
| GQS (0–5) | ChatGPT 5.2 vs Grok 4.1 | 0.25683926 | 1 | -0.428571 |
| GQS (0–5) | ChatGPT 5.2 vs Qwen3-Max | 0.0075263152 | 0.060210521 | 0.833333 |
| GQS (0–5) | DeepSeek-V3.2 vs Gemini 3 Pro | 0.52708926 | 1 | 0.250000 |
| GQS (0–5) | DeepSeek-V3.2 vs Grok 4.1 | 0.70545699 | 1 | -0.142857 |
| GQS (0–5) | DeepSeek-V3.2 vs Qwen3-Max | 0.0080093156 | 0.060210521 | 0.848485 |
| GQS (0–5) | Gemini 3 Pro vs Grok 4.1 | 0.3657123 | 1 | -0.333333 |
| GQS (0–5) | Gemini 3 Pro vs Qwen3-Max | 0.0038924171 | 0.035031754 | 1.000000 |
| GQS (0–5) | Grok 4.1 vs Qwen3-Max | 0.0016162222 | 0.016162222 | 0.866667 |
| ARI | ChatGPT 5.2 vs DeepSeek-V3.2 | 0.95632935 | 0.99601746 | 0.019048 |
| ARI | ChatGPT 5.2 vs Gemini 3 Pro | 0.32998276 | 0.98994827 | 0.261905 |
| ARI | ChatGPT 5.2 vs Grok 4.1 | 0.0055809021 | 0.02790451 | -0.685714 |
| ARI | ChatGPT 5.2 vs Qwen3-Max | 0.0031528473 | 0.018917084 | -0.723810 |
| ARI | DeepSeek-V3.2 vs Gemini 3 Pro | 0.029575348 | 0.11830139 | 0.552381 |
| ARI | DeepSeek-V3.2 vs Grok 4.1 | 0.00032234192 | 0.0022563934 | -0.847619 |
| ARI | DeepSeek-V3.2 vs Qwen3-Max | 3.6239624e-05 | 0.00028991699 | -0.933333 |
| ARI | Gemini 3 Pro vs Grok 4.1 | 1.9073486e-05 | 0.00019073486 | -0.952381 |
| ARI | Gemini 3 Pro vs Qwen3-Max | 2.6702881e-05 | 0.00024032593 | -0.942857 |
| ARI | Grok 4.1 vs Qwen3-Max | 0.49800873 | 0.99601746 | -0.180952 |
| FRES | ChatGPT 5.2 vs DeepSeek-V3.2 | 0.18382534 | 0.55147601 | 0.347368 |
| FRES | ChatGPT 5.2 vs Gemini 3 Pro | 0.2024498 | 0.55147601 | -0.338095 |
| FRES | ChatGPT 5.2 vs Grok 4.1 | 1.9073486e-06 | 1.9073486e-05 | 1.000000 |
| FRES | ChatGPT 5.2 vs Qwen3-Max | 0.00016784668 | 0.0010070801 | 0.880952 |
| FRES | DeepSeek-V3.2 vs Gemini 3 Pro | 0.0019778227 | 0.0079112908 | -0.830409 |
| FRES | DeepSeek-V3.2 vs Grok 4.1 | 5.7220459e-06 | 4.5776367e-05 | 0.980952 |
| FRES | DeepSeek-V3.2 vs Qwen3-Max | 0.00020980835 | 0.0010490417 | 0.866667 |
| FRES | Gemini 3 Pro vs Grok 4.1 | 1.9073486e-06 | 1.9073486e-05 | 1.000000 |
| FRES | Gemini 3 Pro vs Qwen3-Max | 5.7220459e-06 | 4.5776367e-05 | 0.980952 |
| FRES | Grok 4.1 vs Qwen3-Max | 0.70118141 | 0.70118141 | 0.104762 |
| GFI | ChatGPT 5.2 vs DeepSeek-V3.2 | 0.0051561215 | 0.020624486 | -0.731579 |
| GFI | ChatGPT 5.2 vs Gemini 3 Pro | 0.72850609 | 0.72850609 | -0.095238 |
| GFI | ChatGPT 5.2 vs Grok 4.1 | 0.0014324188 | 0.0071620941 | -0.776190 |
| GFI | ChatGPT 5.2 vs Qwen3-Max | 2.6702881e-05 | 0.00024032593 | -0.942857 |
| GFI | DeepSeek-V3.2 vs Gemini 3 Pro | 0.00070762634 | 0.0049533844 | 0.809524 |
| GFI | DeepSeek-V3.2 vs Grok 4.1 | 0.036233902 | 0.10870171 | -0.533333 |
| GFI | DeepSeek-V3.2 vs Qwen3-Max | 0.0010166168 | 0.0060997009 | -0.790476 |
| GFI | Gemini 3 Pro vs Grok 4.1 | 0.00015485726 | 0.0012388581 | -0.989474 |
| GFI | Gemini 3 Pro vs Qwen3-Max | 1.335144e-05 | 0.0001335144 | -0.961905 |
| GFI | Grok 4.1 vs Qwen3-Max | 0.12309265 | 0.2461853 | -0.404762 |
| FKGL | ChatGPT 5.2 vs DeepSeek-V3.2 | 0.5216732 | 1 | -0.171429 |
| FKGL | ChatGPT 5.2 vs Gemini 3 Pro | 0.78412628 | 1 | 0.076190 |
| FKGL | ChatGPT 5.2 vs Grok 4.1 | 0.0001335144 | 0.00093460083 | -0.885714 |
| FKGL | ChatGPT 5.2 vs Qwen3-Max | 0.0004825592 | 0.002412796 | -0.828571 |
| FKGL | DeepSeek-V3.2 vs Gemini 3 Pro | 0.0063896179 | 0.025558472 | 0.676190 |
| FKGL | DeepSeek-V3.2 vs Grok 4.1 | 0.0001335144 | 0.00093460083 | -0.885714 |
| FKGL | DeepSeek-V3.2 vs Qwen3-Max | 4.7683716e-05 | 0.00038146973 | -0.923810 |
| FKGL | Gemini 3 Pro vs Grok 4.1 | 3.8146973e-06 | 3.8146973e-05 | -0.990476 |
| FKGL | Gemini 3 Pro vs Qwen3-Max | 2.6702881e-05 | 0.00024032593 | -0.942857 |
| FKGL | Grok 4.1 vs Qwen3-Max | 0.26109886 | 0.78329659 | -0.295238 |
| CL | ChatGPT 5.2 vs DeepSeek-V3.2 | 0.57059669 | 1 | -0.157143 |
| CL | ChatGPT 5.2 vs Gemini 3 Pro | 0.013616562 | 0.040849686 | 0.619048 |
| CL | ChatGPT 5.2 vs Grok 4.1 | 3.8146973e-06 | 3.0517578e-05 | -0.990476 |
| CL | ChatGPT 5.2 vs Qwen3-Max | 0.0001335144 | 0.00066757202 | -0.885714 |
| CL | DeepSeek-V3.2 vs Gemini 3 Pro | 0.0010166168 | 0.0040664673 | 0.795238 |
| CL | DeepSeek-V3.2 vs Grok 4.1 | 3.8146973e-06 | 3.0517578e-05 | -0.990476 |
| CL | DeepSeek-V3.2 vs Qwen3-Max | 3.8146973e-06 | 3.0517578e-05 | -0.990476 |
| CL | Gemini 3 Pro vs Grok 4.1 | 1.9073486e-06 | 1.9073486e-05 | -1.000000 |
| CL | Gemini 3 Pro vs Qwen3-Max | 1.9073486e-06 | 1.9073486e-05 | -1.000000 |
| CL | Grok 4.1 vs Qwen3-Max | 0.57059669 | 1 | -0.152381 |
| SMOG | ChatGPT 5.2 vs DeepSeek-V3.2 | 0.16495705 | 0.42871857 | -0.361905 |
| SMOG | ChatGPT 5.2 vs Gemini 3 Pro | 0.54587555 | 0.54587555 | -0.166667 |
| SMOG | ChatGPT 5.2 vs Grok 4.1 | 0.0010166168 | 0.0060997009 | -0.790476 |
| SMOG | ChatGPT 5.2 vs Qwen3-Max | 0.0004825592 | 0.0033779144 | -0.828571 |
| SMOG | DeepSeek-V3.2 vs Gemini 3 Pro | 0.10539818 | 0.42159271 | 0.423810 |
| SMOG | DeepSeek-V3.2 vs Grok 4.1 | 0.0042209625 | 0.021104813 | -0.704762 |
| SMOG | DeepSeek-V3.2 vs Qwen3-Max | 0.00039482117 | 0.0031585693 | -0.838095 |
| SMOG | Gemini 3 Pro vs Grok 4.1 | 6.2942505e-05 | 0.00056648254 | -0.914286 |
| SMOG | Gemini 3 Pro vs Qwen3-Max | 1.9073486e-05 | 0.00019073486 | -0.952381 |
| SMOG | Grok 4.1 vs Qwen3-Max | 0.14290619 | 0.42871857 | -0.385714 |

Supplementary Table S7. Reliability scores with mean ± SD and median [Q1, Q3]

| Model | DISCERN total score (16–80) | EQIP total (% yes; 0–100) | JAMA total score (0–4) | GQS (0–5) |
| --- | --- | --- | --- | --- |
| ChatGPT 5.2 | 48.45 ± 6.08 49.00 [45.25, 51.25] | 75.00 ± 7.25 75.00 [70.00, 80.00] | 0.55 ± 0.60 0.50 [0.00, 1.00] | 4.00 ± 0.46 4.00 [4.00, 4.00] |
| DeepSeek-V3.2 | 54.45 ± 6.80 54.50 [51.00, 59.25] | 73.00 ± 5.48 75.00 [70.00, 76.25] | 0.05 ± 0.22 0.00 [0.00, 0.00] | 4.10 ± 0.55 4.00 [4.00, 4.00] |
| Gemini 3 Pro | 53.00 ± 7.43 52.00 [47.75, 58.25] | 76.50 ± 4.89 75.00 [75.00, 80.00] | 0.40 ± 0.68 0.00 [0.00, 1.00] | 4.00 ± 0.46 4.00 [4.00, 4.00] |
| Grok 4.1 | 55.25 ± 8.29 57.50 [51.75, 61.25] | 79.25 ± 6.34 77.50 [75.00, 85.00] | 1.20 ± 0.83 1.00 [0.75, 2.00] | 4.15 ± 0.49 4.00 [4.00, 4.00] |
| Qwen3-Max | 41.85 ± 5.23 43.50 [37.00, 45.25] | 68.00 ± 5.94 67.50 [65.00, 70.00] | 0.30 ± 0.57 0.00 [0.00, 0.25] | 3.50 ± 0.61 4.00 [3.00, 4.00] |

Supplementary Table S8. Readability metrics with mean ± SD and median [Q1, Q3]

| Model | ARI | FRES | GFI | FKGL | CL | SMOG |
| --- | --- | --- | --- | --- | --- | --- |
| ChatGPT 5.2 | 14.30 ± 2.39 14.34 [12.90, 15.75] | 32.95 ± 11.97 30.00 [25.75, 40.50] | 13.79 ± 1.82 14.00 [12.38, 15.50] | 12.53 ± 2.01 12.52 [11.07, 14.16] | 15.18 ± 2.00 15.71 [14.07, 16.66] | 11.06 ± 1.46 10.84 [10.04, 12.19] |
| DeepSeek-V3.2 | 14.25 ± 1.88 14.24 [13.37, 15.12] | 29.75 ± 9.41 30.50 [23.00, 33.25] | 14.96 ± 1.71 15.10 [13.95, 16.20] | 12.97 ± 1.58 13.02 [12.23, 13.71] | 15.41 ± 1.51 15.54 [14.62, 16.68] | 11.58 ± 1.42 11.39 [11.03, 12.29] |
| Gemini 3 Pro | 13.69 ± 1.58 13.64 [12.72, 14.68] | 36.40 ± 6.57 36.00 [32.50, 40.25] | 13.81 ± 1.31 13.75 [13.03, 14.30] | 12.36 ± 1.31 12.19 [11.64, 13.63] | 14.22 ± 1.18 14.32 [13.52, 15.23] | 11.23 ± 1.06 11.05 [10.25, 12.26] |
| Grok 4.1 | 17.00 ± 2.10 16.61 [15.87, 18.05] | 16.95 ± 8.94 18.50 [11.50, 21.25] | 15.89 ± 1.41 15.80 [15.00, 16.60] | 15.26 ± 1.82 15.04 [14.19, 16.25] | 17.75 ± 1.56 17.61 [16.78, 18.46] | 12.88 ± 1.55 12.98 [11.84, 13.73] |
| Qwen3-Max | 17.08 ± 3.04 18.18 [15.01, 19.08] | 17.40 ± 14.01 15.00 [9.00, 20.00] | 16.65 ± 2.35 17.15 [16.48, 18.05] | 15.58 ± 2.72 16.39 [14.75, 17.16] | 17.98 ± 2.32 17.90 [16.84, 19.65] | 13.47 ± 2.12 13.71 [12.14, 15.09] |

Supplementary Table S9. Overall between-model differences (Friedman) with exact P values

| Outcome | Friedman χ² | df | P | Kendall W | P (Holm, across outcomes) |
| --- | --- | --- | --- | --- | --- |
| DISCERN total score (16–80) | 50.061224 | 4 | 3.5061267e-10 | 0.625765 | 2.8049014e-09 |
| EQIP total (% yes; 0–100) | 31.295522 | 4 | 2.6645231e-06 | 0.391194 | 1.0658093e-05 |
| JAMA total score (0–4) | 35.758621 | 4 | 3.2441453e-07 | 0.446983 | 1.6220727e-06 |
| GQS (0–5) | 19.027523 | 4 | 0.00077621854 | 0.237844 | 0.00077621854 |
| ARI | 26.400000 | 4 | 2.6278537e-05 | 0.330000 | 5.2557074e-05 |
| FRES | 50.589421 | 4 | 2.7196641e-10 | 0.632368 | 2.4476977e-09 |
| GFI | 37.648241 | 4 | 1.3242873e-07 | 0.470603 | 7.945724e-07 |
| FKGL | 38.400000 | 4 | 9.2661071e-08 | 0.480000 | 6.486275e-07 |
| CL | 56.120000 | 4 | 1.8923107e-11 | 0.701500 | 1.8923107e-10 |
| SMOG | 29.080000 | 4 | 7.5302474e-06 | 0.363500 | 2.2590742e-05 |

Supplementary Table S10. JAMA item prevalence and total-score distribution

| Model | Measure | Yes_n | Yes_% |
| --- | --- | --- | --- |
| ChatGPT 5.2 | JAMA1 | 0 | 0.0 |
| ChatGPT 5.2 | JAMA2 | 10 | 50.0 |
| ChatGPT 5.2 | JAMA3 | 0 | 0.0 |
| ChatGPT 5.2 | JAMA4 | 1 | 5.0 |
| ChatGPT 5.2 | JAMA total = 0 | 10 | 50.0 |
| ChatGPT 5.2 | JAMA total = 1 | 9 | 45.0 |
| ChatGPT 5.2 | JAMA total = 2 | 1 | 5.0 |
| ChatGPT 5.2 | JAMA total = 3 | 0 | 0.0 |
| ChatGPT 5.2 | JAMA total = 4 | 0 | 0.0 |
| DeepSeek-V3.2 | JAMA1 | 0 | 0.0 |
| DeepSeek-V3.2 | JAMA2 | 1 | 5.0 |
| DeepSeek-V3.2 | JAMA3 | 0 | 0.0 |
| DeepSeek-V3.2 | JAMA4 | 0 | 0.0 |
| DeepSeek-V3.2 | JAMA total = 0 | 19 | 95.0 |
| DeepSeek-V3.2 | JAMA total = 1 | 1 | 5.0 |
| DeepSeek-V3.2 | JAMA total = 2 | 0 | 0.0 |
| DeepSeek-V3.2 | JAMA total = 3 | 0 | 0.0 |
| DeepSeek-V3.2 | JAMA total = 4 | 0 | 0.0 |
| Gemini 3 Pro | JAMA1 | 0 | 0.0 |
| Gemini 3 Pro | JAMA2 | 6 | 30.0 |
| Gemini 3 Pro | JAMA3 | 0 | 0.0 |
| Gemini 3 Pro | JAMA4 | 2 | 10.0 |
| Gemini 3 Pro | JAMA total = 0 | 14 | 70.0 |
| Gemini 3 Pro | JAMA total = 1 | 4 | 20.0 |
| Gemini 3 Pro | JAMA total = 2 | 2 | 10.0 |
| Gemini 3 Pro | JAMA total = 3 | 0 | 0.0 |
| Gemini 3 Pro | JAMA total = 4 | 0 | 0.0 |
| Grok 4.1 | JAMA1 | 0 | 0.0 |
| Grok 4.1 | JAMA2 | 15 | 75.0 |
| Grok 4.1 | JAMA3 | 0 | 0.0 |
| Grok 4.1 | JAMA4 | 9 | 45.0 |
| Grok 4.1 | JAMA total = 0 | 5 | 25.0 |
| Grok 4.1 | JAMA total = 1 | 6 | 30.0 |
| Grok 4.1 | JAMA total = 2 | 9 | 45.0 |
| Grok 4.1 | JAMA total = 3 | 0 | 0.0 |
| Grok 4.1 | JAMA total = 4 | 0 | 0.0 |
| Qwen3-Max | JAMA1 | 0 | 0.0 |
| Qwen3-Max | JAMA2 | 5 | 25.0 |
| Qwen3-Max | JAMA3 | 0 | 0.0 |
| Qwen3-Max | JAMA4 | 1 | 5.0 |
| Qwen3-Max | JAMA total = 0 | 15 | 75.0 |
| Qwen3-Max | JAMA total = 1 | 4 | 20.0 |
| Qwen3-Max | JAMA total = 2 | 1 | 5.0 |
| Qwen3-Max | JAMA total = 3 | 0 | 0.0 |
| Qwen3-Max | JAMA total = 4 | 0 | 0.0 |

Note: In this dataset, JAMA1 and JAMA3 were absent across all responses (0% prevalence), indicating near-zero variance for these items.
